# Surfactant Protein D Binds to *Coxiella burnetii* and Results in a Decrease in Interactions with Murine Alveolar Macrophages

**DOI:** 10.1371/journal.pone.0136699

**Published:** 2015-09-14

**Authors:** Kelly A. Soltysiak, Erin J. van Schaik, James E. Samuel

**Affiliations:** Department of Microbial Pathogenesis & Immunology, Texas A&M Health Science Center, Bryan, Texas, United States of America; University of North Dakota, UNITED STATES

## Abstract

*Coxiella burnetii* is a Gram-negative, obligate intracellular bacterium and the causative agent of Q fever. Infections are usually acquired after inhalation of contaminated particles, where *C*. *burnetii* infects its cellular target cells, alveolar macrophages. Respiratory pathogens encounter the C-type lectin surfactant protein D (SP-D) during the course of natural infection. SP-D is a component of the innate immune response in the lungs and other mucosal surfaces. Many Gram-negative pulmonary pathogens interact with SP-D, which can cause aggregation, bactericidal effects and aid in bacterial clearance. Here we show that SP-D binds to *C*. *burnetii* in a calcium-dependent manner with no detectable bacterial aggregation or bactericidal effects. Since SP-D interactions with bacteria often alter macrophage interactions, it was determined that SP-D treatment resulted in a significant decrease in *C*. *burnetii* interactions to a mouse alveolar macrophage model cell line MH-S indicating SP-D causes a significant decrease in phagocytosis. The ability of SP-D to modulate macrophage activation by *C*. *burnetii* was tested and it was determined that SP-D does not alter the correlates measured for macrophage activation. Taken together these studies support those demonstrating limited activation of alveolar macrophages with *C*. *burnetii* and demonstrate interactions with SP-D participate in reduction of phagocyte attachment and phagocytosis.

## Introduction

*Coxiella burnetii* is a Gram-negative zoonotic bacterial pathogen that is the etiologic agent of Q Fever in humans and coxiellosis in animals [1]. Q fever is usually acquired after contact with infected animals. While infection with *C*. *burnetii* often results in asymptomatic seroconversion, Q Fever can also present as an acute febrile illness, which can either resolve or result in chronic infection most often manifesting as endocarditis [2]. *C*. *burnetii* is subject to an phenomenon that occurs in many Gram-negative bacteria, the transition from “smooth” to “rough” LPS, which occurs after serial passage in a non-immunocompetent host [3]. This transition can occur via the deletion of O-antigen or core carbohydrate biosynthesis genes. *C*. *burnetii* RSA439 a clonal isolate that serves as a model for this phenomenon contains a deletion of a 26 kDa fragment of the genome, which renders it unable to produce O-antigen [4]. Smooth variants, designated phase I are virulent during animal infection, whereas, rough variants designated phase II are attenuated in immunocompetent animal infection. However, both phase I and phase II infect and multiply equally in a variety of cell types [5].

Infection by *C*. *burnetii* occurs via inhalation of aerosolized bacteria into the alveolar space within the lungs where *C*. *burnetii* proceeds to infect and survive within a variety of target cells, predominantly alveolar macrophages. Alveolar macrophages represent a unique lineage due to environmental conditions within the alveolar space [6]. Recent studies conducted with isolated alveolar macrophages from bronchial alveolar lavage have shown that virulent and avirulent strains of *C*. *burnetii* are able to infect alveolar macrophages and stimulate the production of TNF-α, IL-6, and IL-10 [7]. In addition to this alveolar macrophage study, studies using other monocytes or lineage macrophages have demonstrated that infection with either virulent or avirulent *C*. *burnetii* results in the production of TNF-α and IL-6 [5]. Therefore, *C*. *burnetii* infection triggers an early robust pro-inflammatory response.

Pulmonary pathogens are exposed to many components of the innate immune system that are usually involved in clearance of these invading organisms. For example, respiratory pathogens that reach the alveolar space encounter C-type lectins including surfactant proteins, often modeled by surfactant protein D (SP-D) [8]. SP-D is a hydrophilic protein that has been shown to be involved in both pulmonary surfactant homeostasis and the innate immune response [9]. Structurally SP-D is a tetramer composed of homotrimeric subunits that interact via their N-terminal domain, have a collagen-like domain and a C-terminal carbohydrate recognition domain (CRD) [10]. SP-D binds to various self and non-self ligands through its CRD in a calcium-dependent manner and then interacts with immune cells through its collagen-like domain to activate immune cells for clearance of the pathogen [10]. SP-D has been shown to directly interact with a number of bacteria including *Pseudomonas aeruginosa*, *Streptococcus pneumoniae*, *Escherichia coli*, and *Mycobacterium tuberculosis* leading to a number of physiologically relevant processes associated with bacterial clearance including agglutination, phagocytosis, and growth inhibition [11–15]. It is probable that *C*. *burnetii* interacts with SP-D in the lungs before invasion of alveolar macrophages, which may regulate these interactions. In this study, we show that SP-D binds directly to *C*. *burnetii* but does not lead to aggregation or cause cytotoxic affects. In addition, we show that the interaction between SP-D and *C*. *burnetii* modulates bacterial attachment and phagocytosis by alveolar macrophages, but that the level of macrophage activation is not altered.

## Materials and Methods

### Bacteria and cell culture

*C*. *burnetii* RSA439 and RSA493 were grown in either axenic ACCM-2 media or L-929 mouse fibroblasts (ATCC CCL-1) and genome equivalent were determined by real-time PCR, as previously described [16,17]. Briefly, DNA was purified from *C*. *burnetii* at indicated time points using Roche High Pure PCR Template Prep Kit and then 2 uL of purified DNA was used in 20 uL real time PCR reactions using ABI Sybergreen master mix (Applied Biosystems by Life Technologies, Foster City, CA, USA) and primers specific for *com1* (CBU_1910). Prior to use, the *C*. *burnetii* were centrifuged and washed once in PBS to remove residual ACCM-2 or SP Buffer. *Escherichia coli* DH5α were grown in LB broth and quantified using OD_600_ or viable counts. Prior to use, *E*. *coli* were washed once with PBS to remove residual LB. RSA493 experiments were conducted under BSL3 containment according to Texas A&M University Office of Biosafety standard operating procedures. The murine alveolar macrophage cell line (MH-S) [18] were propagated in RPMI supplemented with 1X GlutaMAX (Gibco by Life Technologies, Foster City, CA, USA), 10% heat-inactivated fetal bovine serum, 18 mM sodium bicarbonate, 10 mM HEPES, 25 mM glucose, and 0.05 mM 2-mercaptoethanol at 37 degrees in 5% CO_2_. For *C*. *burnetii* infections MH-S cells were seeded at 1 x 10^5^ per mL and allowed to adhere overnight.

### SP-D binding, agglutination and antimicrobial assays

Binding to SP-D was determined using a previously described solution phase assay with some modifications [19]. Briefly, ~1 x 10^8^ *C*. *burnetii* or *E*. *coli* were incubated with 1mg/mL recombinant human SP-D (R&D Systems Inc, Minneapolis, MN, USA) in PBS + 5 mM CaCl_2_, PBS + 5 mM CaCl_2_ + 0.1 M galactose or PBS + 5 mM CaCl_2_ +10 mM EDTA overnight at 37°C. Bacteria were washed three times with PBS via centrifugation and resuspended in PBS + 10X sample buffer. Samples were briefly boiled and then subjected to 12.5% SDS-PAGE and transferred onto nitrocelluloase membrane at 100 V for 1 hour. The membrane was blocked with 10% powdered milk in PBS and then probed with polyclonal anti-SP-D (My BioSource, San Diego, CA, USA), polyclonal anti-*C*. *burnetii*, or *E*. *coli* antibodies for 1 hour at 37°C. The membranes were washed 3 times with TBS + 0.05% Tween 20 and probed with HRP- goat anti-rabbit secondary antibody (BioRad, Hercules, CA, USA). The protein bands were visualized using chemiluminescence reagent (GE Healthcare Life Sciences, Pittsburgh, PA, USA).

Agglutination assays were performed by incubated ~1 x 10^8^ *C*. *burnetii* or *E*. *coli* with 1mg/mL SP-D in PBS + 5mM CaCl_2_ in a cuvette that remained stationary for the duration of the assay. At selected time points the OD_600_ of the bacterial suspension was measured.

Growth inhibition assays were performed using 1 x 10^3^ *E*. *coli* or 1 x 10^5^ *C*. *burnetii*. *E*. *coli* were treated with 10mg/mL SP-D for 1 hr at 37°C and then subjected to viability counts. *C*. *burnetii* were treated with 10mg/mL SP-D for 12 hrs at 37°C. *C*. *burnetii* suspensions were added ACCM-2 and incubated at 37°C in a microaerophilic incubator at 2.5% O_2_ and 5% CO_2_. Every two days, 1 ml bacterial culture was removed and DNA was extracted and quantified via real-time PCR as previously described [16].

### Attachment and Phagocytosis assay

MH-S cells were seeded at 1 x 10^5^ cells per ml onto glass cover slips in 24 well tissue culture plates. MH-S cells were infected with CFSE-stained (Molecular Probes by Life Technologies, Foster City, CA, USA) *C*. *burnetii* at MOI 100 and incubated at 37°C. At the indicated time post infection, media was removed and MH-S cells were washed 3 times with RPMI. Coverslips were then fixed with 2% para-formaldehyde and the nuclei were stained with Hoescht (Molecular Probes by Life Technologies, Foster City, CA, USA). Infected MH-S cells were visualized using a Nikon-A1 confocal microscope with 450 and 488 nm filters using the 60X oil immersion objective. A minimum of 100 nuclei from MH-S cells were counted and CFSE *C*. *burnetii* were enumerated within those cells. The infectivity index was calculated as the number of infected cells (bound and internalized *C*. *burnetii*) divided by the total number of counted cells, multiplied by 100.

### Cytokine production

MH-S cells were infected with *C*. *burnetii* at an MOI of 100 and at the indicated times post infection, RNA was extracted from MH-S cells using QiaShredder columns (Qiagen, Valencia, CA, USA) followed by RNEasy kit (Qiagen, Valencia, CA, USA) treatment according to manufacturer’s protocol. Residual DNA was removed using DNA-free kit (Ambion, by Life Technologies, Foster City, CA, USA). RNA was then quantified using a nanophotometer (Implen Inc, Westlake Village, CA, USA) and 500 ng was applied to each reverse transcription reaction using TaqMan Reverse Transcription kit (Applied Biosystems by Life Technologies, Foster City, CA, USA). After reverse transcription, 2.5 uL was used for real-time PCR using primers listed in Table 1. The fold change in expression for each gene was determined using the 2^-ΔCt^ method and murine *gapdh* as a reference [20]. Average *gadph* Ct values among all samples did not deviate more than ± 1 Ct over the 3 independent experiments run with each sample in triplicated to verify reproducibility of results and to comply with MIQE standards [21]. Student t-tests were conducted to determine significance. Nitrite production in MH-S culture supernatant was determined using Griess reagents (Invitrogen by Life Technologies, Foster City, CA, USA) according to the manufacturer’s protocol.

**Table 1:** Real-time polymerase chain reaction primers used in this study.

Primer	Sequence
*Il1b* F	GCA ACT GTT CCT GAA CTC AAC T
*Il1b* R	ATC TTT TGG GGT CCG TCA ACT
*Il4* F	TTT GAA CGA GGT CAC AGG AG
*Il4* R	TTC TTC GTT GCT GTG AGG AC
*Il6* F	TAG TCC TTC CTA CCC CAA TTT CC
*Il6* R	TTG GTC CTT AGC CAC TCC TTC
*Il10* F	GCT CTT ACT GAC TGG CAT GAG
*Il10* R	CGC AGC TCT AGG AGC ATG TG
*Il12b* F	CCA GAG ACA TGG AGT CAT AG
*Il12b* R	AGA TGT GAG TGG CTC AGA GT
*Csf2* F	CAG CTT CTC AGA CTG CTG CT
*Csf2* R	CTT GGT CCC TTT AAG GCA GA
*Tnfa* F	CCC TCA CAC TCA GAT CAT CTT CT
*Tnfa* R	GCT ACG ACG TGG GCT ACA G
*Nos2* F	GTT CTC AGC CCA ACA ATA CAA GA
*Nos2* R	GTG GAC GGG TCG ATG TCA C
*Gapdh* F	TGT GTC CGT CGT GGA TCT GA
*Gapdh* R	CCT GCT TCA CCA CCT TCT TGA

## Results

### SP-D binds *C*. *burnetii* in a calcium-dependent manner, but does not cause aggregation or inhibition of growth

**Fig 1:**
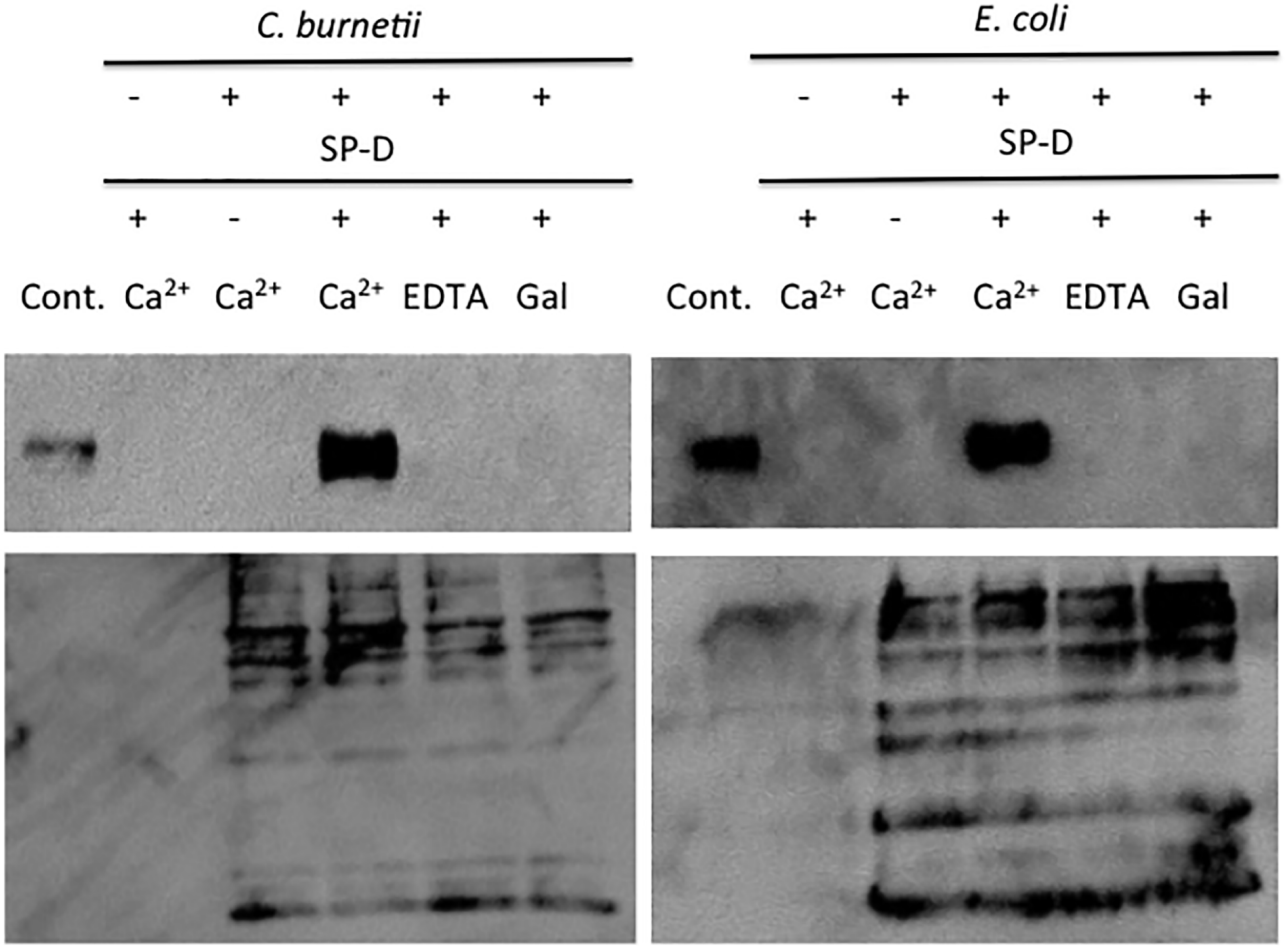
SP-D binds to *C*. *burnetii* in a calcium dependent fashion that can be inhibited by monosaccride. SP-D was incubated with *C*. *burnetii* or *E*. *coli* with or without EDTA or galactose, in PBS + CaCl_2_ overnight at 37°C. Bacterial suspensions were then washed 3x with PBS by centrifugation. All samples were subjected to SDS-PAGE followed by blotting to detect SP-D that co-sedimented with the bacteria. SP-D was loaded as a positive control (left lane each blot labeled SP-D). Representative results are shown from 3 independent experiments.

**Fig 2:**
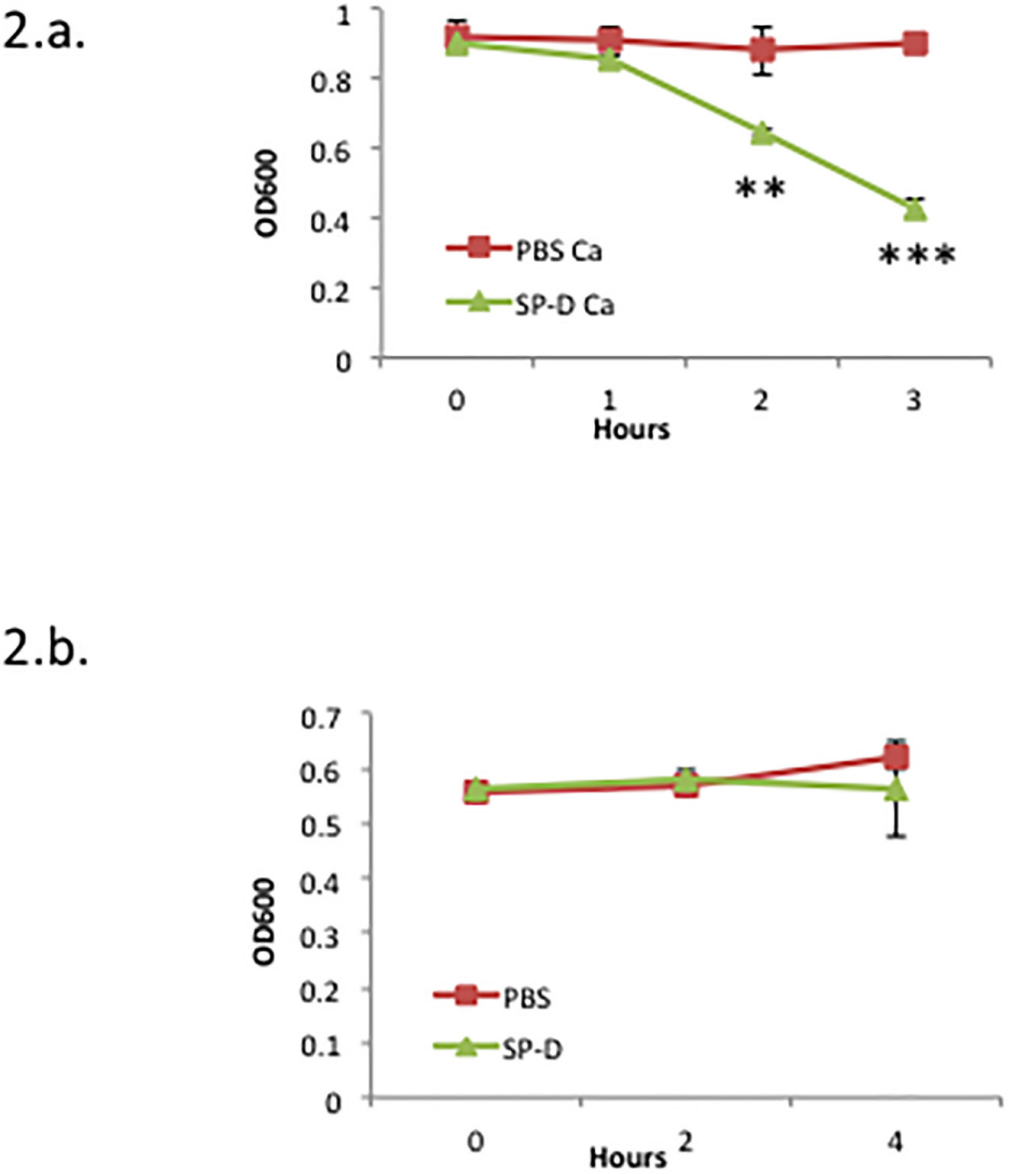
SP-D does not cause aggregation of *C*. *burnetii*. (A) *E*. coli or (B) *C*. *burnetii* were incubated with or without SP-D in PBS + CaCl_2_ in an undisturbed suspension for 3 or 4 hrs, respectively. The ability of SP-D to aggregate the bacteria was determined using spectrometry readings at OD_600_. The data are representative of 3 independent experiments ± standard deviation. Student t-tests compare data sets +/- SP-D ** p<0.005, *** p<0.0005.

**Fig 3:**
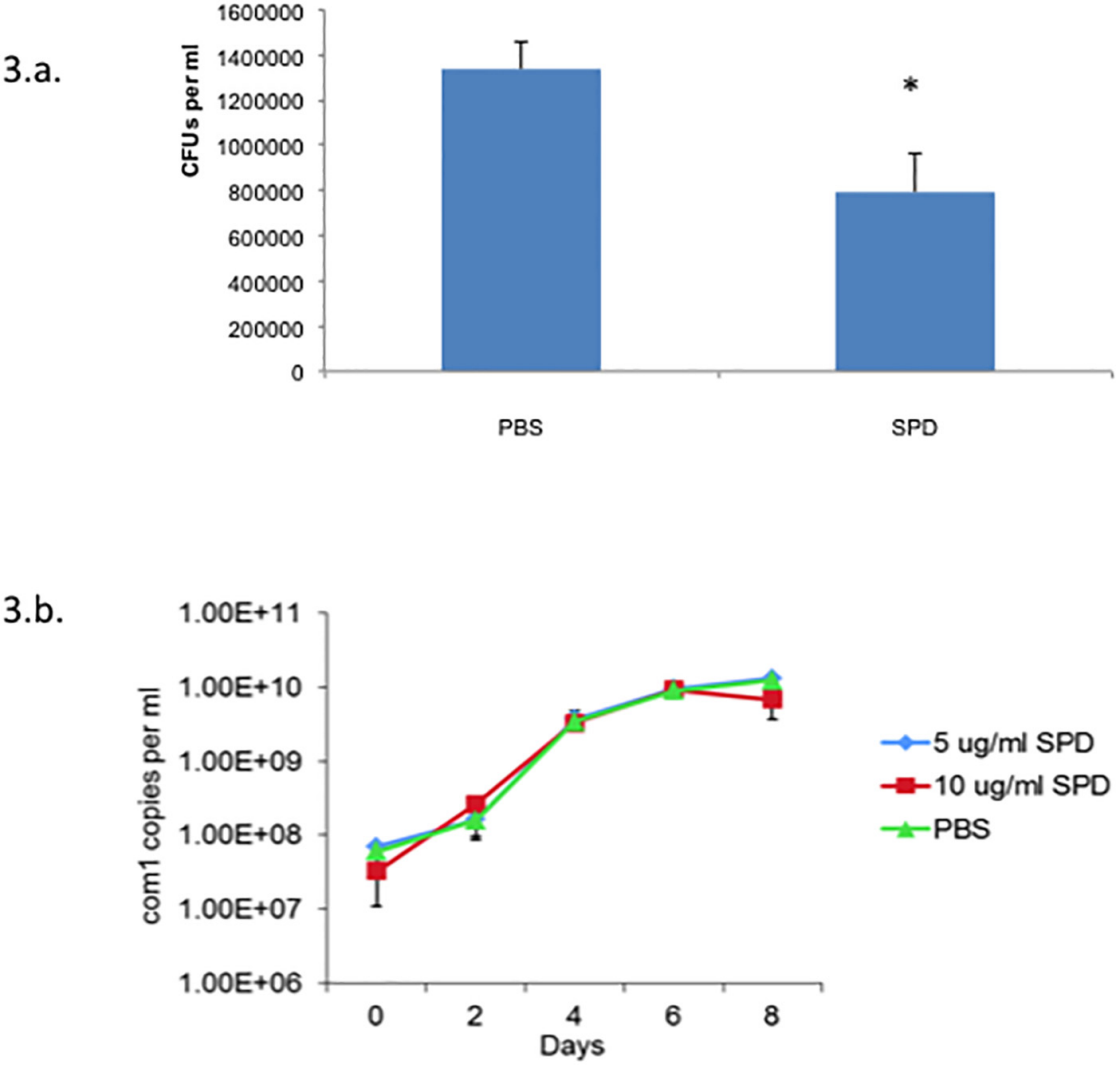
SP-D is not bactericidal towards *C*. burnetii. (A) *E*. *coli* were treated with or without SP-D for one hour at 37°C followed by viable counts on LB. Data displayed are mean ± standard error of three independent experiments. Student t-test compares SP-D to PBS treated *E*. *coli*. * p<0.05. (B) *C*. *burnetii* were treated with 0 (triangles), 5 (squares) or 10 mg/ml (squares) SP-D overnight and transferred to ACCM-2. Every 2 days genome equivalents (GE) were calculated by real-time PCR. Data displayed are mean ± standard deviation of GE/mL, representative of three independent experiments.

To determine if SP-D binds to *C*. *burnetii*, we used a solution phase assay. As shown in Fig 1, SP-D bound to *C*. *burnetii*. The addition of EDTA, a calcium chelator, abolished the interaction between *C*. *burnetii* and SP-D, which confirms that the binding was calcium dependent, a hallmark of most SP-D interactions (Fig 1). In addition, the interaction between SP-D and *C*. *burnetii* was inhibited in the presence of galactose, a monosaccharide with a high binding affinity for SP-D (Fig 1) [22]. The ability of SP-D to aggregate *C*. *burnetii* was determined using a spectrophotometry assay. SP-D was able to aggregate *E*. *coli* in agreement with previous results, however, SP-D was unable to aggregate *C*. *burnetii* (Fig 2a and 2b) [14]. To determine if SP-D was bactericidal towards *C*. *burnetii* we incubated the bacteria with SP-D and the ability to replicate was monitored in ACCM-2. SP-D did not significantly effect the viability of *C*. *burnetii*, however, incubation with SP-D did significantly decrease the viability of *E*. *coli* in agreement with previous results (Fig 3a and 3b) [14].

### SP-D causes a decrease in *C*. *burnetii* interactions with macrophages

**Fig 4:**
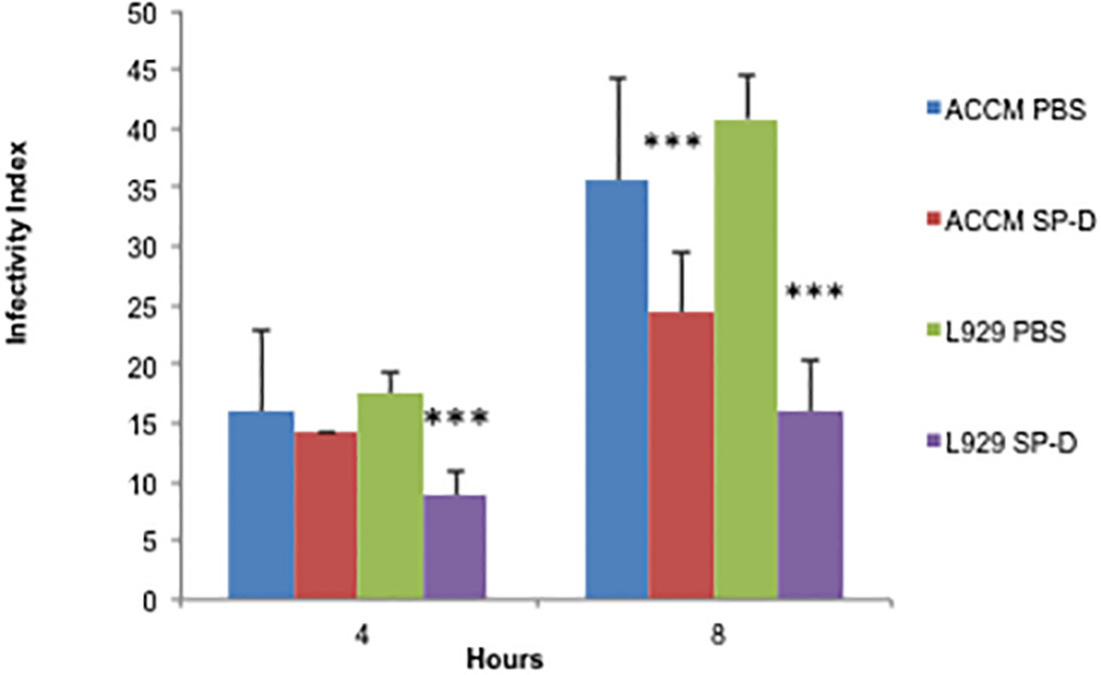
SP-D treatment results in a decrease in infectivity. ACCM-2 or L-929 passaged *C*. *burnetii* were stained with CSFE and treated overnight with SP-D or the equivalent volume of buffer alone (PBS). MH-S cells were infected at MOI 100 for 4 or 8 hours and fixed. MH-S nuclei were stained with Hoescht, the number of infected cells and bacteria was quantified via confocal microscopy, and the infectivity index was calculated (bound and internalized *C*. *burnetii*). Data are displayed as mean ± standard deviation of infectivity from one representative of 5 experiments, n = 3. Student t-tests compare SP-D to PBS treated groups. *, p<0.05, ***, p<0.0005.

SP-D interactions are known to modulate attachment and uptake of many Gram- negative bacteria by a variety of cell types [11,23,24]. To determine whether SP-D was able to modulate the adherence and uptake of *C*. *burnetii* by MH-S cells, a murine alveolar macrophage cell line, the amount of adherent and internalize *C*. *burnetii* was determined using fluorescently labeled bacteria [18]. We used both *C*. *burnetii* grown in axenic ACCM-2 media and *C*. *burnetii* grown in L-929 cells to determine if growth in axenic media alters the physiological properties of the bacterium. As shown in Fig 4, SP-D treatment significantly reduced the *C*. *burnetii* infectivity index (bound and internalized *C*. *burnetii*) at 8 hrs of infection for both ACCM-2 and L-929 grown bacteria. Interestingly, we observed that SP-D was able to modulate interactions of L-929 grown *C*. *burnetii* at 4 hrs, but had no effect at this time for ACCM-2 grown *C*. *burnetii* (Fig 4). This suggests that the passage history (media vs cell line infection) of the *C*. *burnetii* may affect binding interactions with cells via surface receptor that interact with the ligand SP-D.

### SP-D treatment does not result in modulation of macrophage activation

**Fig 5:**
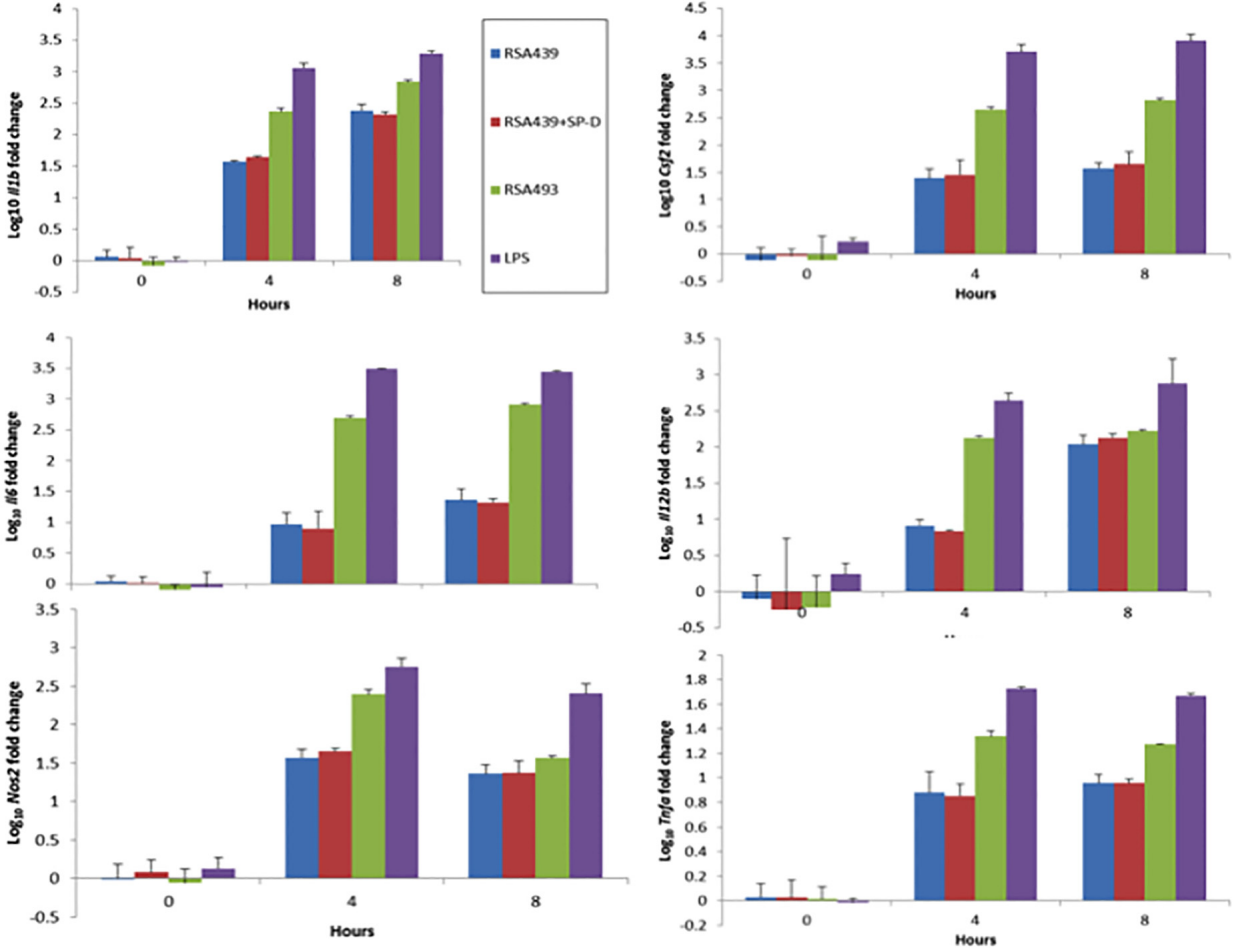
SP-D treatment does not alter *C*. *burnetii* stimulated transcriptional patterns in MH-S cells. MH-S cells were infected with PBS or SP-D treated *C*. *burnetii* RSA439 (phase II), RSA493 (phase I) or treated with *E*. *coli* LPS for 0, 4, and 8 hours. RNA was extracted as described and applied to real-time PCR. Data represent the mean ± standard deviation of fold change in RNA expression of select genes compared to expression of host cell *Gapdh*, n = 3. RSA439 and LPS data are representative of three independent experiments, RSA493 data are representative of two independent experiments. Student t-tests compare each time and condition to its corresponding 0 time point, all RSA439 and RSA439 + SP-D were significantly up-regulated * p<0.05. There was no significant difference in expression between untreated and SP-D treated *C*. *burnetii* RSA439 as determined by student t-tests.

**Fig 6:**
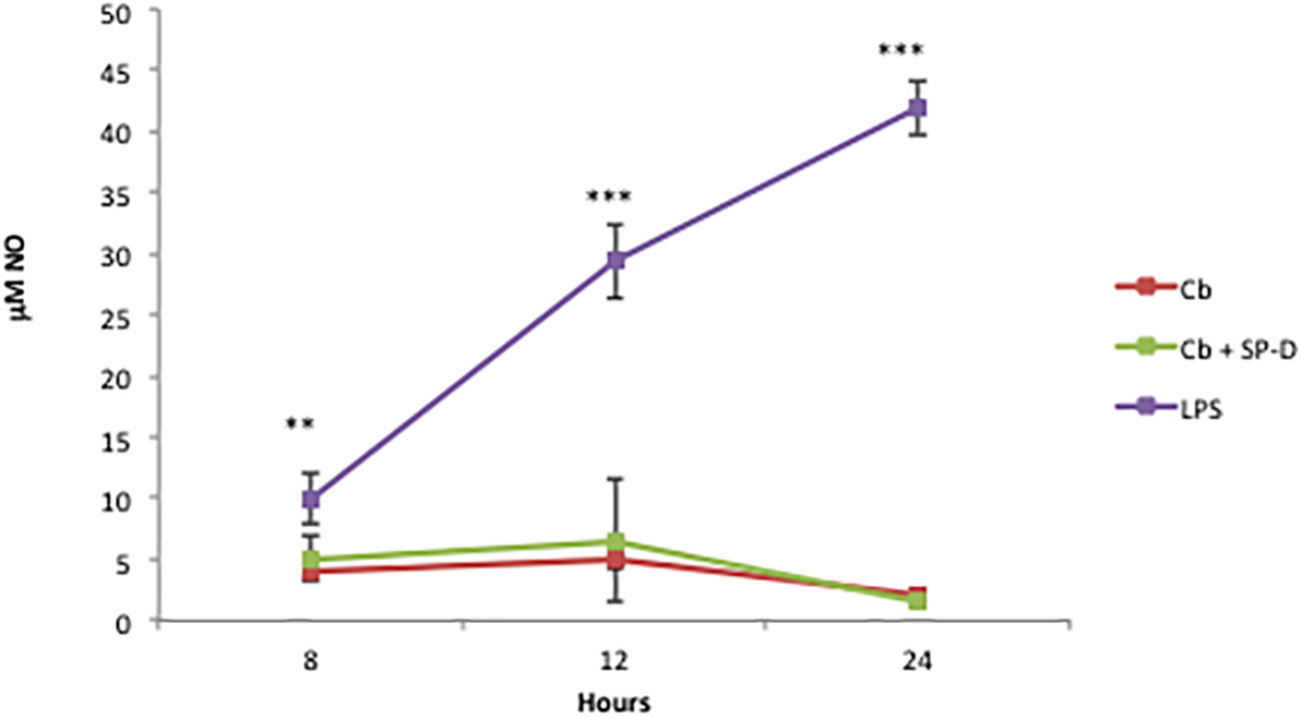
SP-D treatment does not alter *C*. *burnetii* stimulated nitric oxide secretion. MH-S cells were infected with PBS or SP-D treated *C*. *burnetii* (Cb or Cb + SP-D respectively) or *E*. *coli* LPS (LPS) for 8, 12, and 24 hours at which point cell supernatants were removed and evaluated for nitrate production via the Greiss assay. The data represent the mean ± standard deviation of NO mM in cell culture supernatant n = 3 and are compared to uninfected and untreated controls using student t-tests ** p<0.005, *** p<0.0005.

Our goals were two-fold in the comparison of *C*. *burnetii* infection of this alveolar macrophage line. First we wanted to determine if MH-S cells mimic the activation observed by human alveolar macrophages due to *C*. *burnetii* infection to assess their usefulness as model cell line and then to determine if SP-D alters this activation [7]. MH-S cells have been used as an *in vitro* model for *Legionella pneumophila* infection the most closely related bacterial species to *C*. *burnetii* as an alternative to hard to obtain primary alveolar macrophages [25,26]. Therefore, we determined the transcriptional responses of MH-S cells to both virulent phase I and avirulent phase II *C*. *burnetii*. The following genes were selected to evaluate macrophage activation: *Il1b*, *Il4*, *Il6*, *Il10*, *Il12b*, *Cfs2*, *Nos2* and *Tnfa*. *E*. *coli* lipopolysaccharide (LPS) was used as a positive control for macrophage activation. As shown in Fig 5, MH-S infected with either phase I or phase II *C*. *burnetii* resulted in a significant induction of *Il1b*, *Il6*, *Il12b*, *Csf2*, *Nos2* and *Tnfa* when compared to uninfected control. Infection with *C*. *burnetii* did not induce significant production of *Il4* or *Il10* (Fig 5). Although infection with *C*. *burnetii* increase the expression of *Nos2* there was no production of nitrites by these cells, which agrees with previous results using human (Fig 6) [27]. Pre-incubation of phase II *C*. *burnetii* with SP-D prior to infection did not affect the transcriptional response of MH-S cells (Fig 6).

## Discussion

Our data demonstrate that *C*. *burnetii* interacts with SP-D in a calcium-dependent manner that can be inhibited by carbohydrate. SP-D has been previously shown to bind to the LPS of many diverse pathogenic Gram-negative bacteria such as *Pseudomonas aeruginosa*, uropathogenic *E*. *coli*, *Klebsiella pneumoniae* and *Helicobacter pylori* in a calcium-dependent manner that can be inhibited by carbohydrate [11,19,28,29]. Furthermore, it was determined *C*. *burnetii* interactions with SP-D did not cause bacterial agglutination or bactericidal effects. Most bacterial pathogens that bind SP-D are also agglutinated by SP-D including *Streptococcus pneumoniae*, *Mycobacterium tuberculosis*, *H*. *pylori* and approximately 50% of clinical *Pseudomonas aeruginosa* isolates [12,13,30,31]. However, other examples of SP-D binding without aggregation exist, such as with *Chlamydia trachomatis* [32]. Thus the interactions of *C*. *burentii* with SP-D do not result in aggregation similar to *C*. *trachomatis*. Bactericidal effects of SP-D have only been demonstrated for *E*. *coli* or rough LPS variants of other bacteria such as *Bordetella pertussis* [14,33]. Therefore, it is somewhat surprising that phase II *C*. *burnetii*, which is a rough variant is resistant to the bactericidal effects of SP-D.

Interactions with SP-D can also modulate adherence and phagocytosis; therefore, we investigated the effects of SP-D on *C*. *burnetii* interactions with MH-S cells. We observed that SP-D decreases the infectivity of *C*. *burnetii* to MH-S cells (bound and internalized bacteria). It is most likely that a decrease in adherence is responsible for this phenotype, but since this study did not distinguish between bound and internalized *C*. *burnetii* that remains to be determined. These interactions most closely resemble those observed for *M*. *tuberculosis* where SP-D decreases adherence and therefore phagocytosis [30]. Other outcomes of SP-D interaction include increased adherence to macrophages but decreases phagocytosis as for *Pneumocystis carinii* or increased phagocytosis as seen with *C*. *trachomatis* [24,32]. Alternatively, SP-D can cause increased uptake and killing of *P*. *aerugionsa* and *K*. *pneumoniae* [11,34].

Our data also indicate that *C*. *burnetii* infection drives MH-S alveolar macrophages towards classical activation and the resulting transcriptional patterns are not altered with SP-D treatment. Several other studies have evaluated the effects of SP-D on bacterial activation in macrophages. SP-D has no effect on the secretion of TNFα induced by *P*. *aeruginosa* [35]. On the other hand, SP-D coated *K*. *pneumophila* cause an increase in expression of IL-6, IL-10, and IL-12 [36]. LPS induces classical macrophage activation that results in resistance to intracellular pathogens and is characterized by secretion of IL-1β, TNF, IL-12, and IL-6 [27]. The activation patterns between *C*. *burnetii* and *E*. *coli* LPS were similar, although the magnitude of induction was different hence the conclusion of classic activation. Interestingly, we observed a greater induction of cytokine expression for phase I *C*. *burnetii* versus phase II *C*. *burnetii* at very early time points (4 and 8 hrs) after infection. This is in contrast to the cytokine release profiles observed after 24 hours of infection in human derived alveolar macrophages, where phase II causes the largest release of cytokines [7]. These differences may be due to the time points observed or due to analyzing expression profiles over cytokine release. The expression profiles after infection of *L*. *pneumophila* in MH-S cells and human derived alveolar macrophages were found to be similar [26]. We therefore conclude that MH-S cells represent a viable model for studying *C*. *burnetii* alveolar macrophage interactions.

In conclusion, SP-D binding to *C*. *burnetii* is calcium-dependent and can be inhibited by carbohydrate. SP-D decreases the interactions of *C*. *burnetii* to MH-S cells, these interactions are most similar to *M*. *tuberculosis*. Future experiments will help define the role of SP-D in the innate response to *C*. *burnetii*. For example, it will be important to determine if SP-D coated *C*. *burnetii* are viable once phagocytosed or if coated of SP-D causes intracellular killing as it does for the closely related *L*. *pneumophila* [37].
